# Modern Sociology

**Published:** 1898-12-17

**Authors:** 


### Modern Sociology.

### THE LIQUOR TRADE IN AMERICA.

### I.—Consumption.

The liquor question being among the things that are ever with us, it may be of use to give some account of of the trade in intoxicants in that country which is most akin to our own, even though the points of difference outnumber those of resemblance. The chief points of difference are the habits of the Americans in regard to drinking, and the heroic measures which are taken to control the evil of drunkenness. To an Englishman, indeed to a European of any nation, the drinking customs of the United States seem so strange and unsocial that he cannot reconcile himself to them, while they fully explain the conviction which is almost universal among American women, that "drinking" and "drunkenness" are convertible terms. Save among those people whom their harsh compatriots call "anglomaniacs," wine or beer is not taken at meals. In an American hotel, as everyone has noticed, you will see a hundred persons going through an elaborate dinner with the accompaniment of iced water. Neither is there the Continental custom of the café or the beer-garden, where a family will sit and drink a modest quantity of some mild fermented beverage, "without fear and without reproach." On the outside, the Americans seem to be the most abstemious of nations; and it requires experience to tell one that they make up for this by "bolting" drinks of the most varied, and often of the most unwholesome, description before meals. It remains to be seen whether this makes more for sobriety than our usages.

The Commissioners of Labour appointed by the Government of the United States have recently devoted a volume to the conditions of the liquor traffic. While this volume deals primarily with the economic effects of the trade, it cannot fail to throw some interesting sidelights on the moral effects of it also. It appears, first, that the average consumption of intoxicants per head is fairly large, considering the very earnest efforts made all over the United States to promote total abstinence. In 1896 the per capita consumption stood at 16·42 gallons. When we add that the consumption has risen rapidly during the last fifty years—for in 1840 it was only 4·17 gallons per head—it would seem as if the Americans were on the high road to become a nation of drunkards. But the figures must be analysed. The increase has been entirely in the consumption of beer, which is perhaps the most innocuous of all fermented liquors. In other liquors there has been a decided falling off. Thus in 1840 the consumption of spirits was about 2½ gallons per head, while in 1896 it had sunk to 1 gallon. Wines were consumed at the rate of 0·29 per head at the earlier date, and 0·26 at the later. A marked decrease in the consumption of spirits began in 1880, and has continued with but slight fluctuations ever since; but it is worth noting that between 1888 and 1893, while the consumption of wine went down, that of spirits showed a tendency to rise, though it never got beyond 1½ gallons per head annually, thus keeping well below the average for 1840, at which date our available statistics begin. It is evident, therefore, that while the Americans are drinking more liquor, they are consuming a milder kind.

During all this time the consumption of beer has shown a steady and large increase. This implies a growing liking for some refreshing and light stimulant; but this is not wholly, though no doubt it is in part, due to any change in the tastes of what, accurately speaking, we can call the American people. Germans are the largest beer consumers in the world, and during the last 30 years there has been an enormous influx of Germans into the United States. They form now by far the largest number of immigrants, and the addition of so many Germans to the population of the States would of itself account for great part of the increased consumption of beer, apart from any influence their example might have on native-born Americans, or the tendency of their children to follow their fathers' example.

It is rather remarkable that the consumption of wines has not increased of late years, since America itself has become a wine-producing country. Perhaps the use of home, *i.e.*, American-made wines, has interfered with the importation of foreign ones. Certainly the importation of wines has not increased during the last 50 years. In 1840 it was 4,748,362 gallons, and in 1896 it was only 4,101,649 gallons. This, even, was an increase of a million gallons or so over the consumption of 1895; while it is to be noted that from 1860 to 1870 the average annual consumption of foreign wines was about nine million gallons. Thus while the consumption of wines per head of the population has varied very little, it is obvious that the new consumers have largely used wines made in their own country. This is shown by the fact that while in 1840 the consumption of American wines was only 124,734 gallons, in 1896 it stood at 14,599,757 gallons. And this was by no means the largest figure that domestic wines had to show. In 1888 their consumption stood at the figure of 31,680,523 gallons, since which time it has been diminishing along with that of spirits. All this time the consumption of beer has risen steadily. This seems to imply that while absolute abstinence is less universal than it was half a century ago, temperance is much more common. Not so many people, in proportion to a largely increased population, eschew every form of intoxicating liquor, but far more avoid those which contain a considerable proportion of alcohol. Teetotalism is less common; but so also is drunkenness. American literature of a comparatively recent date shows always the two extremes. There was the severe puritan type which looked upon the man who took a modest glass of beer with his dinner (to correct the superabundance of pie) as foredoomed to a drunkard's grave; and there was the literature which showed the miner celebrating the find of a pocket of gold, or the death (by revolver or otherwise) of a comrade, by endless drinking of the rawest whisky. Both these types seem to be vanishing from literature, as from life. The temperance crusade does not grow weaker; but it takes a larger view. Drunkenness is held in greater abhorrence than before; but there is no longer the same conviction that no one can drink the mildest of fermented liquors without falling into absolute drunkenness. In the increasing consumption of beer in the United States, combined, as it is, with a decrease in the consumption of all other liquors, we may read the fact that the Americans are beginning to appreciate the value of something that will stimulate digestion without tempting to bad habits, something more harmless to the digestion than candy, and less harmful to the moral nature than the drugs which are apt to become a substitute for intoxicants.

